# Altered protease and antiprotease balance during a COPD exacerbation contributes to mucus obstruction

**DOI:** 10.1186/s12931-015-0247-x

**Published:** 2015-07-15

**Authors:** Shashi Chillappagari, Jenni Preuss, Sebastian Licht, Christian Müller, Poornima Mahavadi, Gaurav Sarode, Claus Vogelmeier, Andreas Guenther, Lutz Nahrlich, Bruce K. Rubin, Markus O. Henke

**Affiliations:** Department of Pediatrics, Justus Liebig University Giessen, Feulgenstrasse 12, Giessen, 35392 Germany; Department of Internal Medicine, Justus-Liebig-University Giessen, Klinikstrasse-36, Giessen, 35392 Germany; Department of Pulmonary Medicine, Philipps-University Marburg, Baldingerstrasse 1, Marburg, 35043 Germany; Department of Pediatrics, Virginia Commonwealth University School of Medicine, 1001 East Marshall Street, Richmond, 23298 VA USA; Asklepios Fachkliniken München-Gauting, Robert-Koch-Allee 2, Gauting, 82131 Germany; Member of the Comprehensive Pneumology Center (CPC), Helmholtz Zentrum, Munich, Germany; Member of the German Centre for Lung Research (DZL), Giessen, Germany; Member of the European IPF Registry/Biobank, Giessen, Germany; Lung Clinic Waldhof-Elgershausen, Greifenstein, Germany

**Keywords:** COPD, Mucin, Proteases, Alpha-1-protease inhibitor, Neutrophil elastase, Cigarette smoke, Hypersecretion

## Abstract

**Background:**

Proteases have been shown to degrade airway mucin proteins and to damage the epithelium impairing mucociliary clearance. There are increased proteases in the COPD airway but changes in protease-antiprotease balance and mucin degradation have not been investigated during the course of a COPD exacerbation. We hypothesized that increased protease levels would lead to mucin degradation in acute COPD exacerbations.

**Methods:**

We measured neutrophil elastase (NE) and alpha 1 protease inhibitor (A1-PI) levels using immunoblotting, and conducted protease inhibitor studies, zymograms, elastin substrate assays and cigarette smoke condensate experiments to evaluate the stability of the gel-forming mucins, MUC5AC and MUC5B, before and 5–6 weeks after an acute pulmonary exacerbation of COPD (*n* = 9 subjects).

**Results:**

Unexpectedly, mucin concentration and mucin stability were highest at the start of the exacerbation and restored to baseline after 6 weeks. Consistent with these data, immunoblots and zymograms confirmed decreased NE concentration and activity and increased A1-PI at the start of the exacerbation. After recovery there was an increase in NE activity and a decrease in A1-PI levels. *In vitro,* protease inhibitor studies demonstrated that serine proteases played a key role in mucin degradation. Mucin stability was further enhanced upon treating with cigarette smoke condensate (CSC).

**Conclusion:**

There appears to be rapid consumption of secreted proteases due to an increase in antiproteases, at the start of a COPD exacerbation. This leads to increased mucin gel stability which may be important in trapping and clearing infectious and inflammatory mediators, but this may also contribute acutely to mucus retention.

## Introduction

Proteases play a major role in bacterial entrapment [5], pathogen phagocytosis [16], mucin hypersecretion and mucociliary clearance [9]. In COPD there is a deficiency and decreased activity of anti-proteases [21, 30], contributing to emphysema [1] and mucus hypersecretion [4]. This protease and anti-protease imbalance has been suggested to result from neutrophil infiltration in the lung [3, 28]. These neutrophils release proteases including neutrophil elastase (NE), cathepsin-G (CG), and proteinase 3 (PR3).

Mucins are linearly linked core proteins encoded by mucin (MUC) genes. The principal airway gel-forming mucins are MUC5AC and MUC5B [24]. Several lines of evidence show that mucus is hypersecreted in COPD [30]. Studies performed on surgically isolated lung tissues from COPD patients have shown that mucus containing inflammatory exudate accumulates in small airways and is associated with disease progression [11]. In biopsies from COPD patients with severe lung disease, mucus occupies about 15 % of the total luminal area of small airways; whereas, in “healthy” smokers, it is limited to less than 5 % of the luminal area [13]. The amount of small airway mucus is strongly associated with mortality in patients with COPD [12]. We have shown that increased secretion of serine proteases in cystic fibrosis (CF) can degrade the gel-forming mucins during the time of transport from peripheral airways to central airways [9]. However the effect of serine proteases on mucin in COPD subjects has not been well characterized.

In this study, we investigated the role of proteases and anti-proteases on COPD mucin stability and degradation during the course of an infectious and inflammatory exacerbation of COPD. As in subjects with CF, we hypothesized that that there would be an acute increase in proteases during exacerbation leading to mucin degradation.

## Methods

### Subject details and sample collection

Nine subjects were included in the study, with a mean age of 59.9 years. They had been hospitalized or evaluated in the outpatient clinic of the Department of Pulmonary Medicine, Philipps-University Marburg, because of an acute pulmonary exacerbation of COPD defined by the Anthonisen criteria of increased dyspnea and cough, increased sputum volume and change in sputum color [2]. Subjects were included if they had at least 2 of symptoms with an onset within 7 days before the start of the study. We included only subjects in GOLD group II or III (FEV1/VC < 70 %, FEV1 30–80 %) [31]. Criteria for exclusion were signs of bacterial infection with fever >38.5 °C, CRP-elevation > 30 mg/L or procalcitonin elevation > 5 μg/L, suspected or known pneumonia with infiltrate on chest x-ray, *Pseudomonas aeruginosa* in sputum cultures, pre-admission antibiotic treatment, or suspected or known asthma. At the first day of the reported pulmonary exacerbation symptoms, sputum was collected. All subjects were followed up 5–6 weeks after the onset of the exacerbation and another sputum sample was collected (Table 1). At visit 1 the subjects were grouped as “COPD with exacerbation” and after 5–6 weeks (visit 2) as “COPD without exacerbation”. All subjects were treated with oral steroids (40 mg once daily) for total of 10 days, and inhalation therapy with long-acting muscarinic antagonists and short- and long-acting beta2-agonists. Five of the 9 subjects were current smokers and 4 were former smokers. Antibiotic treatment was not necessary for any of the subjects and all of them recovered from the exacerbation within the observed time. Clinical characteristics and demographics of the COPD subjects are given in Table 2. Sputum collection was approved by the Philipps-University Marburg Institutional Review Board.

**Table 1:** Study summary

	Time	Procedures
Visit 1	day 1; week 1 (within 7 days after start of exacerbation)	Sputum collection, pulmonary function test, chest x-ray, blood sample, physical examination
Visit 2	days 40–46, week 5–6	Sputum collection, pulmonary function test, blood sample

**Table 2:** Demographic data of the COPD patients included the study

subject	age	Pack years	smoking status		chest x-ray (infiltrations?)	CRP in mg/l	leucocytes in G/l (normal: 4.3–10)	Procalcitonin in μg/l	FEV1 (%)	VC (%)	FEV1/VC (%)	color of sputum
01	51	80	current smoker	Visit 1	no	<5	6.24	*	49	95	47	clear
				Visit 2		<5	7.05	*	58	94	55	clear
02	61	80	former smoker	Visit 1	no	<5	8.28	0.22	63	95	66	clear/slightly yellow
				Visit 2		14	8.14	0.17	67	95	70	clear
03	74	20	former smoker	Visit 1	unkown	31	7.25	*	67	95	70	clear
				Visit 2		18	6.64	*	81	95	75	clear
04	67	20	former smoker	Visit 1	no	11	10.7	<0.1	48	89	53	clear/slightly yellow
				Visit 2		*	*	*	42	98	43	clear
05	65	30	former smoker	Visit 1	no	18	3.92	<0.1	68	98	69	clear
				Visit 2		<5	5.21	<0.1	95	98	97	clear
06	52	50	current smoker	Visit 1	no	11	13	*	32	93	34	clear
				Visit 2		<5	9.15	*	74	98	75	clear
07	57	35	current smoker	Visit 1	no	<5	5.89	*	37	91	41	clear
				Visit 2		*	*	*	58	90	64	clear
08	50	50	current smoker	Visit 1	no	14	15.3	<0.1	36	93	39	clear
				Visit 2		24	15.3	<0.1	51	92	55	clear
09	62	70	current smoker	Visit 1	no	7	8.5	*	56	89	63	clear/slightly yellow
				Visit 2		<5	6.15	*	67	89	75	clear

*Data not collected

### Control mucus collection

As a control group we collected mucus coating the endotracheal tubes (ETT) of subjects who had no lung disease and required non-thoracic surgery under general anesthesia. When the subject was extubated, the ETT was removed from the airway and mucus was removed by gently scraping the ETT [25, 26]. Collected ETT mucus was placed in a small O-ring container to prevent dehydration, labeled as to date of collection with no subject identifiers, and sent to Philipps-University Marburg on dry ice. ETT mucus collection was approved by the Virginia Commonwealth University Institutional Review Board and signed consent, and assent when appropriate, was obtained.

### Protease inhibitors and antibodies

NE and cathepsin G were purchased from Merck Chemical, Nottingham, UK. Serine protease inhibitors diisopropyl fluorophosphates (DFP), phenylmethyl sulfonyl fluoride (PMSF), and 1-chloro-3-tosylamido-7-amino-2-heptanone HCl (TLCK), metalloprotease (EDTA and GM6001) and cysteine proteases (leupeptin and E64) were purchased from Sigma (Saint Louis, MO). Alpha-1 protease inhibitor (A1-PI) was obtained as Prolastin® (Grifols Therapeutics Inc. Frankfurt, Germany) and was used at a final concentration of 0.3 μg/mL. DFP (final concentration 2 mM); PMSF (final concentration 2 mM); TLCK (final concentration 10 mM); EDTA (final concentration 100 mM); E64 (final concentration 500 ng/mL) or Merck Chemical (Nottingham, UK): GM6001 (final concentration 40 μM) and leupeptin (final concentration 40 μM) were used. Polyclonal anti-MUC5AC and anti-MUC5B antibodies were generated as previously described [10]. The antibodies were characterized and specificity was ascertained by pre-absorption studies using increasing concentrations of the antigenic peptides [25]. Specificity of these antibodies was verified using immunoblotting against MUC5AC and MUC5B from whole cell lysates, secretions from normal human tracheobronchial epithelial (NHBE) cells (passage 2) (Clonetics Corp., La Jolla, CA, USA), and human mucus. The blots were analyzed with antisera for MUC5AC and MUC5B and the pre-immune sera of the same rabbit. We found one well-defined band of high molecular weight with the antisera. To increase the specificity of the antibodies and reduce nonspecific binding, affinity purification of the antipeptide antibody was performed from the whole serum using the immobilized amino acid sequences of interest (SulfoLink-Kit, Pierce). An internal control for mucin was collected from a voluminous sputum sample from a single patient undergoing lung transplantation for non-CF bronchiectasis [10]. Mucin signals obtained from COPD sputum and normal controls were normalized to this internal control, which was set to 100 %.

### Agarose wet western blotting for MUC5AC and MUC5B

Sputum and internal control samples were diluted 1:10 with PBS and denatured using Laemmli buffer (125 mM Tris pH 6.8; 4 % SDS; 20 % glycerol; 0.001 % bromophenol blue, 20 mM DTT) and separated using 1 % agarose gels (15 × 15 cm), prepared in running buffer (25 mM Tris, 250 mM glycine, 0.1 % SDS). Electrophoresis was performed in a horizontal gel apparatus at 60 V at room temperature for the first 30 min, and then voltage was set to 100 V for the rest of the time. To identify small proteins that remained in the gel, the gel was stopped when the dye front was 2/3 of the distance from the wells. Proteins were transferred to nitrocellulose membranes using vertical wet electroblotting apparatus, LKB bromma at (300 mA) for 3 h at 4 °C. Membranes were blocked with 10 % nonfat skimmed milk in PBS for 1 h and subsequently incubated with primary antibodies (1:100 MUC5AC and 1:100 MUC5B) for 18 h in 1 % nonfat skimmed milk in PBS at 4 °C. Blots were washed 3 times in PBS for 10 min, and incubated with the secondary HRP-labeled goat-anti-rabbit antibody (1:1000) (Jackson-Immuno) in 1 % nonfat skimmed milk in PBS for 1 h. Blots were washed in PBS for 10 min 3 times and developed using the Pico-Developer-Kit (Pierce). Exposures were taken on CL-XPosure film (Pierce) at equal exposure times. The films were scanned and band intensities were determined by densitometry using NIH Image software (http://rsbweb.nih.gov/nih-image/).

### SDS PAGE western blotting for alpha-1-protease inhibitor (A1-PI) NE

Sputum and internal control samples were diluted (1:100 for A1-PI and 1:20 for NE) with PBS and homogenized using a syringe. As a positive control, human A1-PI (Prolastin®) and a control subject sample known to contain NE were used. Upon denaturation, the samples were separated by SDS-PAGE (7.5 % for A1-PI and 10 % for HNE) before blotting onto PVDF membranes. The membranes were blocked with 5 % nonfat skimmed milk in TBST for 1 h at room temperature and incubated with primary antibodies anti- A1-PI (Sigma Aldrich) HNE- (Abcam) over night at 4 °C. Membranes were washed three times in TBST for 15 min, and incubated with the HRP-labeled secondary antibodies dissolved in TBST containing 5 % milk, for 1 h at room temperature. Membranes were developed using ImmunoCruz Luminol agent (Santa Cruz). Exposures were taken on CL-XPosure film (Pierce) at equal exposure times. The film was scanned and band densities were determined by densitometry using NIH Image software (http://rsbweb.nih.gov/nih-image/).

### Analyzing NE activity with specific substrates

To analyze free NE activity in the sputum we used the substrate N-Methoxysucuccinyl -ALA-ALA-PRO -VAL P-Nitroanilide (Sigma Aldrich, Saint Louis, MO). According to the manufacturer, the substrate is specifically hydrolyzed by HNE and cannot be hydrolyzed by cathepsin G. To get comparable results, we used a test volume set at 1000 μL, consisting of 900 μL substrate solution and 5 μL of patient sputum samples with added buffer, adding the enzymatically active compound at last. The test solution was thoroughly mixed and the analysis was started immediately. As an internal control we used triplets of each sample in dilution steps of 1:10, 1:20 and 1:40 in PBS. As an external control 3 μL of purified NE (Calbiochem ®, product no. 324681) was used. The degradation of the substrate was analyzed at a wavelength of 410 nm over 30 min in a Nicolet Evolution 100 UV–vis Spectrophotometer. The results were documented via VISIONlife™ software.

### Analysis of protease activity using zymograms

NOVEX 4–16 % zymogram blue casein gels (Life Technologies) were used to detect NE enzymatic activity in sputum samples. Mucin samples were homogenized using a sterile Filtropur syringe filter (0.20 μm pore size). Equal volumes of homogenized sputum samples were loaded on a gel and separated using electrophoresis at 125 V for 2 h. The gel was run in Tris/glycine SDS running buffer under nondenaturing conditions. The separated proteins were renatured using a buffer containing a non-ionic detergent (Novex® Zymogram Renaturing Buffer). Gels were washed twice for 15 min in PBS and equilibrated using a developing buffer (Novex® Zymogram Developing Buffer) containing divalent metal cations for 30 min as described in the manufacturer’s protocol. The gel was then incubated at 37 °C for 20 h in fresh developing buffer for enhanced digestion. Enzymatic activity was visualized as a clear band against a dark background of stained casein. ETT mucus from healthy subjects was used as a positive control. The gels were scanned using a Canon ScanLide 50 scanner and activity was measured by quantification of digested area using Image-J densitometry software.

### Preparation of cigarette smoke condensate (CSC)

Cigarettes were smoked in a smoking chamber for 5 min and smoke was suctioned using a vacuum pump into a Falcon tube containing 37 °C pre-warmed 10 mL PBS. Care was taken to maintain constant temperature (37 °C) and continuous stirring to allow the smoke to dissolve fully in PBS. One cigarette in 10 mL PBS is referred to as CSC10, which was considered to be 10 %.

### Inhibition of NE-activity by CSC

Inhibition studies of NE were performed spectrophotometrically using specific NE-substrate (Merck Chemicals) as described in the manufacturer protocols (Elastin). Different concentrations of CSC (CSC5, CSC10) were used to inhibit pure NE (final concentration 0.33 μg/ml). Activity of NE was measured at 410 nm.

### Data analysis

All analyses were performed at least in triplicate. Results are presented as mean values ± standard error. The mucin concentration of the sputum samples was normally distributed within all groups (Skewness < ± 2). The mucin concentration is shown as % to an internal control. To compare sputum samples from the same group (COPD week 1 and week 5–6) we used the paired *t*-Test. To compare sputum samples of the different groups we used the Mann–Whitney *U*-Test. After *post hoc* correction for multiple comparisons, a probability of *p* < 0.05 was considered significant. All analyses were performed by means of GraphPad Prism 5 software (San Diego, CA). Descriptive statistics were used to summarize subject demographics.

## Results

### Increase in mucins concentration before exacerbation

**Fig. 1:**
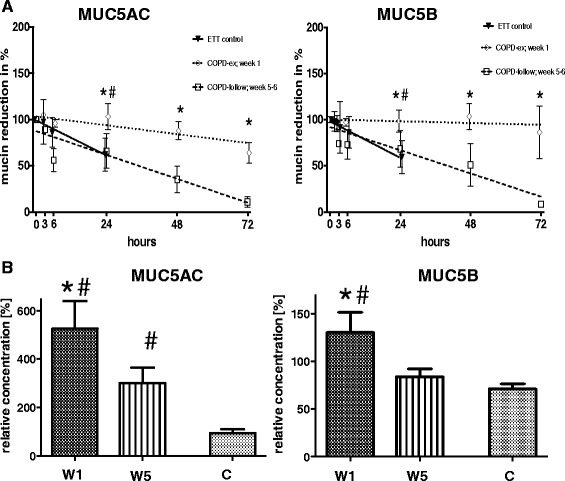
Sputum collection and mucin quantification from COPD subjects. Quantification of mucin in sputum obtained from 9 subjects with COPD. **a** Sputum was collected at the start of an exacerbation of COPD (COPD-ex; week 1) and again 5–6 weeks later (COPD-follow; week 5–6) from the same subjects. Results were compared to mucin concentration in mucus from 11 ETT control mucus samples (control). **b** The results are shown as mean density of individual samples related to the internal control (=100 % relative concentration). * = significant to “COPD-follow; week 5-6” (*t*-Test, *p* < 0.05); # = significant to “control” (Mann Whitney test, *p* < 0.05)

Sputa were collected at the onset of a COPD exacerbation and 5–6 weeks later and compared with ETT mucus obtained from healthy subjects. Mucin stability in samples was analyzed *in vitro* after incubation at 37 °C for 24 h. In comparison to ETT mucus, there was a 5-fold increase in MUC5AC and 2-fold increase in MUC5B at the start of an exacerbation. Five to 6 weeks later, MUC5AC was about 3-fold higher in comparison to ETT control mucus or 2-fold lower than at the start of the exacerbation. MUC5B concentration decreased to ETT mucin levels at 5–6 weeks (Fig 1a, b). Results obtained from immunoblot densitometry showed about a 40 % degradation of sputa from COPD patients at 5–6 weeks with almost no degradation seen at the start of an exacerbation. These observations suggest that there was dramatically (and unexpectedly) increased antiprotease activity or decreased NE activity at the start of the exacerbation, but by week 5, protease and antiprotease activity returns to baseline.

### A1-PI and free NE concentration in sputum

**Fig. 2:**
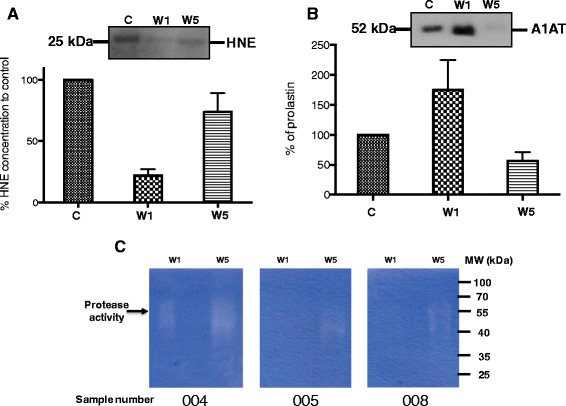
Altered NE and A1-PI in COPD sputum samples. **a** Representative western blot analysis for NE from 3 COPD subjects (003, 005, 007) during the course of exacerbation. C = Control, W1 = week 1, W5 = week 5–6. To compare the results graphically western blots were analyzed by densitometry and compared to an internal control sputum sample, which was set to 100 %. **b** Representative western blot for A1-PI from 3 COPD subjects (003, 005, 007) C = control; W1 = week 1 and W5 = week 5–6. To compare the results graphically western blots were analyzed by densitometry and compared to an internal control sputum sample, which was set to 100. **c** Analysis of sputum protease activity by zymogram. Sputum was obtained from 3 COPD subjects with an acute exacerbation within the first week (COPD-ex; week 1) and from the same subjects 5–6 weeks later (COPD-follow; week 5–6) *n* = 3

In order to understand the reasons for increased mucin stability at the start of an exacerbation, we quantified NE and A1-PI in sputum samples using an immunoblot and found that the NE concentration in the sputum of the COPD patients at the onset was 3.5 times lower than at 5–6 weeks after the onset of the exacerbation (Fig 2a). Additionally, we analyzed nonspecific protease activity in sputa from 3 subjects with COPD at the start of an exacerbation and compared it to sputa from the same subjects 5–6 weeks later using zymograms (Fig. 2c). Dornase alfa was added to the sputum to release proteases trapped in DNA. The strongest signal for nonspecific enzyme activity was detected only in the sputum samples obtained 5–6 weeks after the onset. We found that A1-PI concentration in the sputum of the COPD patients at the beginning of the exacerbation was 3 times higher when compared with sputum 5–6 weeks after the onset of the exacerbation (Fig. 2b). Thus at the start of a COPD exacerbation it appears that protease activity is low which as well correlates to increased A1-PI.

### Increase in A1-PI at the start of an exacerbation inhibits mucin degradation

**Fig. 3:**
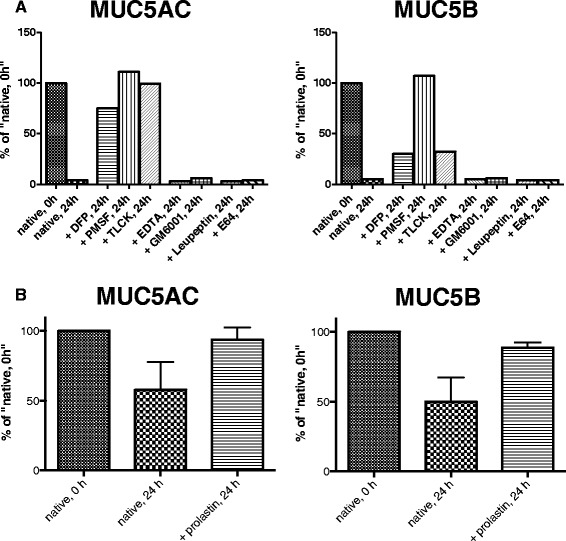
Serine proteases inhibit mucin degradation. Analysis of sputum MUC5AC and MUC5B by western blot after incubation at 37 °C over 24 h with or without of protease inhibitors. Sputum was obtained from a COPD subject 5–6 weeks after an acute exacerbation. Mucin concentration of the native control without incubation over 24 h was set to 100 %. **a** We used the serine protease inhibitors DFP, PMSF and TLCK, the metalloprotease inhibitors EDTA and GM6001 and the cysteine protease inhibitors leupeptin and E64. Analysis was performed in triplicate. **b** Incubation of COPD sputa (5–6 weeks after the onset) with A1-PI (*n* = 4) and compared with control sputa

To verify the role of non-specific proteases in COPD mucin degradation 5–6 weeks after the onset of an exacerbation, we incubated the mucus with different protease inhibitors at 37 °C for 24 h. Results obtained from immunoblots revealed that without protease inhibitors, MUC5AC concentration decreased by 96 % and MUC5B by 95 % of the initial concentration, whereas incubation with the protease inhibitors DFP, PMSF and TLCK inhibited mucin degradation. However, incubation with the metalloprotease inhibitors, EDTA and GM6001, and cysteine protease inhibitors, Leupeptin and E64, did not inhibit the mucin degradation (Fig. 3a). We also tested if A1-PI could decrease mucin degradation. We incubated COPD sputa from 4 subjects at week 5–6 after exacerbation, with and without A1-PI and inhibited the degradation of MUC5AC to just 6 % (SEM ± 9) and MUC5B to 11 % (SEM ± 3) of the native mucin concentration (Fig. 3b).

### Inaccessibility of NE decreases mucin degradation

**Fig. 4:**
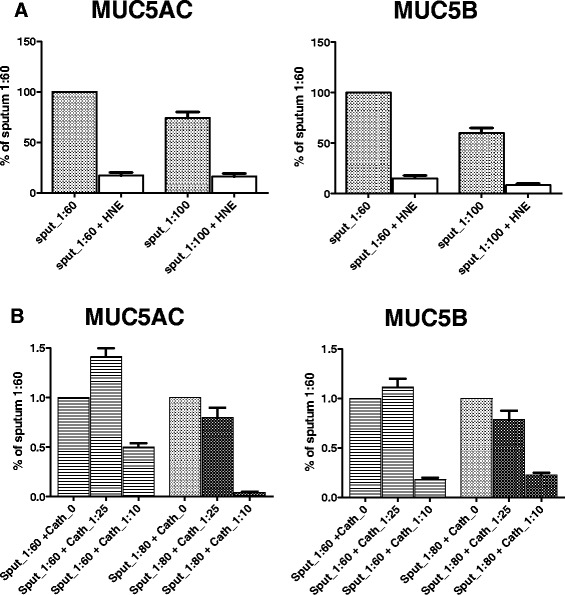
Mucus hydration increases mucin degradation. **a** Sputum was obtained at the onset of a COPD exacerbation and diluted 1:60 and 1:100. Mucin degradation was measured after incubation for 6 h at 37 °C with or without NE 0.02 mg/mL. Mucin concentration of the control at 1:60 was set to 100 %. **b** Sputum was obtained at the onset of a COPD exacerbation and diluted 1:60 and 1:80. Mucin degradation was measured after incubation for 6 h at 37 °C with cathepsin G 100 μU/μL diluted 1:25 and 1:10. Mucin concentration of the control was set at 1:60 and 1:80 was set to 100 %

Serial dilutions of mucus identified that a 1:60 to 1:100 dilution of mucus is most effective for measuring protease activity (Fig. 4a). We then incubated COPD sputum from the start of an exacerbation at dilutions of 1:60 and 1:80 with synthetic proteases, NE 0.02 mg/mL, and cathepsin G 100μU/μL and incubated at 37 °C for 6 h. Both MUC5AC and MUC5B mucins were degraded by NE and cathepsin G in a concentration dependent manner (Fig. 4b).

### Cigarette smoke condensate (CSC) decreases mucin degradation and inhibits protease activity

**Fig. 5:**
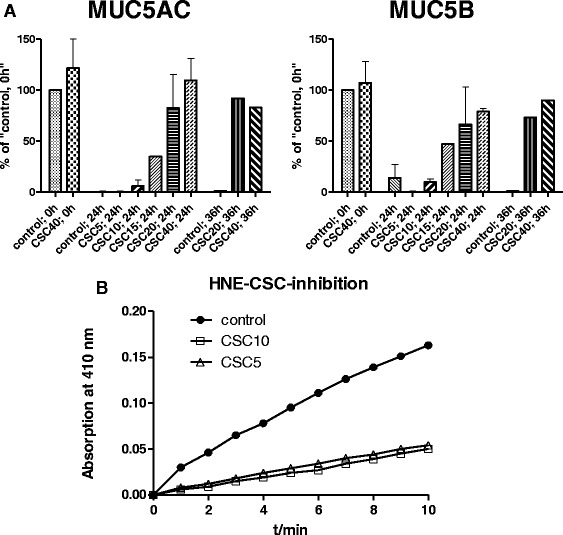
Cigarette smoke extract inhibits NE and increases mucus accumulation. **a** Quantification of COPD mucin degradation after incubation at 37 °C with increasing concentrations of CSC (5–40 mg/mL) for 0, 24 and 36 h. **b** Substrate specific activity of NE 0.33 μg/mL with and without CSC (5–40 mg/mL) . Mucin concentration of the native control was set to 100 %

To analyze the role of CSC on mucin degradation, COPD sputum was incubated with different CSC concentrations (5–40 %) for 0, 24 and 36 h. COPD sputa without CSC, MUC5AC and MUC5B mucins were significantly degraded after 24 and 36 h. A dose dependent inhibition of mucin degradation was observed with COPD sputum incubated with increasing concentrations of CSC (Fig. 5a). To elucidate the role of CSC in inhibiting mucin degradation, we incubated HNE (0.33 μg/mL) with different concentrations (5–10 %) of CSC and analyzed the activity of NE using a HNE specific substrate (5-methoxy-Ala-Ala-Pro-Val). To conclude, CSC directly interfered with protease activity in a dose dependent manner (Fig. 5b).

## Discussion

We had anticipated that with an acute exacerbation of COPD, there would be increased inflammation and increased proteases with subsequent degradation of mucins, Thus we were surprised to discover that at the start of a COPD exacerbation there was consistently decreased proteases, increased anti-proteases, and increased mucin stability. A possible explanation for this is that although we evaluated subjects and collected sputa at the start of *symptoms* of an exacerbation, it is likely that the inciting infection and inflammation had been present for several days. These data might reflect the natural host immune response to decrease the initially observed increased protease activity. Although this is entirely speculative, it would explain these paradoxical results.

One consequence of inhibiting mucin degradation might be increased mucus obstruction, which is considered a hallmark of a COPD exacerbation. It has been reported that persons with COPD have increased mucus synthesis and secretion, and decreased mucus clearance [19, 20, 22]. We have reported that in CF sputum, serine proteases degrade mucins after secretion [9]. Bacterial or host inflammatory cell proteases in CF sputum may further contribute to mucin degradation [23]. Delayed mucin degradation in COPD could well be caused by this protease-anti protease imbalance. We report a 5-fold increase in MUC5AC and a 2 fold increase in MUC5B at the onset of symptoms and even 5–6 weeks later, MUC5AC was 3 times greater in COPD sputa compared to mucus from healthy controls (Fig. 1a, b).

COPD is an inflammatory disease of small airways with increased neutrophil infiltration and NE [14, 30]. Studies performed by other groups suggested that in mucoid COPD sputum no NE was found (NE nM 0.0) [8, 27]. These observations are similar to our findings, where little or no NE activity was observed in mucoid sputa of COPD subjects at the onset of an exacerbation (Fig. 2a, c). In CF, sputum NE is predominantly bound by DNA and this inhibits proteolytic activity [17]. Much of the DNA in CF sputum originates from neutrophil extracellular traps (NETs). It is speculated that NE-NET formations are reservoirs of active proteases for a possible later release [6]. In CF and COPD sputa the DNA concentration is higher than in normal airway secretions, therefore this NE that is bound to DNA is not available for mucin degradation [9, 10, 18]. A1-PI is increased during infection and inflammation and its primary role is to inhibit NE. Consistent with our results (Fig. 2b), during an acute exacerbation of COPD, A1-PI is elevated in sputum [29], serum [7] and in exhaled breath condensate [15]. We did not detect increased NE concentrations or protease activity during an acute COPD exacerbation, which is in agreement with previous studies [8, 27, 32].

We also show that CSC can inhibit mucin degradation in a dose dependent manner by inhibiting the activity of NE (Fig. 5b). Thus tobacco smoke and increased DNA might contribute to mucus retention in COPD.
